# Mitochondrial haplogroup B increases the risk for hearing loss among the Eastern Asian pedigrees carrying 12S rRNA 1555A>G mutation

**DOI:** 10.1007/s13238-015-0203-z

**Published:** 2015-09-11

**Authors:** Zhengbiao Ying, Jing Zheng, Zhaoyang Cai, Li Liu, Yu Dai, Juan Yao, Hui Wang, Yinglong Gao, Binjiao Zheng, Xiaowen Tang, Yi Zhu, Min-Xin Guan, Ye Chen

**Affiliations:** Department of Otolaryngology, Wenling People’s Hospital, Wenzhou Medical University, Wenling, 317500 China; Institute of Genetics, School of Medicine, Zhejiang University, Hangzhou, 310058 China; Department of Clinical Laboratory, The Second Affiliated Hospital, School of Medicine, Zhejiang University, Hangzhou, 310009 China; Attardi Institute of Mitochondrial Biomedicine, School of Laboratory Medicine and Life Sciences, Wenzhou Medical University, Wenzhou, 325035 China; Department of Otolaryngology, The First Affiliated Hospital, Wenzhou Medical University, Wenzhou, 325000 China; Collaborative Innovation Center for Diagnosis and Treatment of Infectious Diseases, Zhejiang University, Hangzhou, 310058 China

**Dear Editor,**

Hearing loss is one of the major public health problems. The mitochondrial DNA mutations has been found to be associated with both aminoglycoside-induced and nonsyndromic hearing loss (Fischel-Ghodsian, 1999; Guan, 2011). The 1555A>G mutation in mitochondrial small subunit ribosomal RNA (rRNA) gene contributes to nonsyndromic hearing loss and is prevalent in Asian and other populations (Guan, 2011). This mutation was first identified in families with aminoglycoside-induced deafness, and the ototoxic susceptibility was attributed to enhanced binding affinity to aminoglycosides of 1555A>G rRNA (Qian and Guan, 2009). In the absence of aminoglycoside antibiotics, individuals with 1555A>G mutation exhibit various clinical phenotype that ranges from severe congenital deafness, through moderate progressive hearing loss of late onset, to completely normal hearing (Lu et al., 2010a). The precise pathology remains unclear; however, it is now largely accepted that genetic susceptibility factors exist and may interact with environmental exposures, leading to the development of hearing loss (Chen et al., 2015; Guan, 2011).

Recent studies have suggested that 1555A>G mutation is of multiple origins, and occurred sporadically among Eastern Asian and European lineages (Lu et al., 2010b). In addition, there are increasing evidence indicated that mtDNA haplogroups defined by distinct sets of mtDNA variants may confer genetic resistance against or susceptibility to certain diseases such as visual failure, diabetes and Parkinson disease (Fuku et al., 2007; Hudson et al., 2007; Khusnutdinova et al., 2008). In this study, we aimed to investigate whether mtDNA haplogroups confer susceptibility to hearing loss in Eastern Asia population carrying 1555A>G mutation.

Comprehensive physical evaluation were performed among 11 probands and members of these Han Chinese pedigrees carrying 1555A>G mutation, and the results revealed that hearing impairment was the only clinical presentation. As shown in Fig. S1, 37 of the 107 matrilineal relatives exhibited bilateral hearing impairment with variable severity ranged from profound, severe, moderate to mild hearing loss, and 11 subjects had a history of aminoglycoside exposure. In addition, variable penetrance of hearing loss were observed among these pedigrees, ranged from 12.5% to 100.0%, with an average of 41.0%. When aminoglycoside-induced hearing loss was excluded, the penetrance of hearing loss in these pedigrees ranged from 0% to 100.0%, with the average of 26.0%.

Through mutational analysis of complete mtDNA genome, we identified a total of 192 variants in mtDNA among the 11 probands, including 48 polymorphisms in D-loop region, 23 variants in 12S rRNA and 16S rRNA, 6 variants in tRNA genes, 75 silent variants and 36 missense mutations in protein encoding genes, and 3 polymorphisms in non-coding region (Table S1). Furthermore, according to their distinct clusters of mtDNA polymorphisms, we found the mtDNAs of 11 pedigrees belonged to 4 common eastern Asian mtDNA haplogroups A, B, D and F.

Besides the 11 families with hearing loss above, we collected additional 131 pedigrees carrying 1555 A>G mutation among eastern Asian population from previous publications for further analysis. Of the 142 mtDNAs analyzed, 74 were categorized into macro-haplogroup M, and the rest 68 pedigrees’ mtDNAs were assigned to macro-haplogroup N. mtDNA haplogroup distribution analysis showed that 1555A>G mutation was widely dispersed among 11 major Eastern Asian haplogroups D, M7, M8, M10, M11, G, F, B, R, A and N9 (Fig. S2 and Table S2). This observation strongly indicated that the 1555A>G mutation arising independently in different mtDNA haplogroup backgrounds among eastern Asian populations. As shown in Table 1, the haplogroup D was most prevalent in pedigrees with 1555A>G mutation, which was significantly higher than control group (*OR* = 2.10; 95% CI = 1.38–3.25; *P* < 0.01). Furthermore, the increased frequency of 1555A>G mutation on haplogroup D background is attributable largely to specific subhaplogroup D5 (*OR* = 2.92; 95% CI = 1.52–5.61; *P* < 0.01). In contrast, those families on haplogroup M8 and G background tended to have a reduced frequency of 1555A>G mutation (*P* < 0.05).

**Table 1:** **Distribution and frequency of mtDNA haplogroup in 1555A>G carriers and controls from Eastern Asia**

Haplogroup	Hearing-impaired subjects (%)	Controls (%)	*P* Value^a^	*OR* (95% CI)^c^
M	74 (52.1)	176 (46.8)	0.3244	
** D**	**52 (36.6)**	**81 (21.5)**	**0.0007** ^**b**^	**2.10 (1.38–3.21)**
D4	30 (21.1)	58 (15.4)	0.1486	
**D5**	**20 (14.1)**	**20 (5.3)**	**0.0015**	**2.92 (1.52–5.61)**
M7	10 (7.0)	26 (6.9)	1.0000	
**M8**	**5 (3.5)**	**40 (10.6)**	**0.0085**	**0.31 (0.12–0.79)**
M9	0 (0)	6 (1.6)	0.1594	
M10	1 (0.7)	5 (1.3)	1.0000	
M11	1 (0.7)	2 (0.5)	1.0000	
**G**	**4 (2.8)**	**16 (4.3)**	**0.0341**	**0.29 (0.095–0.89)**
N	68 (47.9)	200 (53.2)	0.3244	
A	9 (6.3)	25 (6.6)	1.0000	
R	48 (33.8)	142 (37.8)	0.4157	
B	22 (15.5)	70 (18.6)	0.4417	
B4	14 (9.9)	46 (12.2)	0.5389	
B5	7 (4.9)	22 (5.9)	0.8341	
F	23 (16.2)	60 (16.0)	1.0000	
F1	8 (5.6)	32 (8.5)	0.3566	
F2	7 (4.9)	19 (5.1)	1.0000	
F3	4 (2.8)	5 (1.3)	0.2664	
N9	10 (7.0)	32 (8.5)	0.7187	
N9a	7 (4.9)	30 (8.0)	0.2575	
Total subjects	142	376		

^a^ *P* values were calculated by Chi-square test or Fisher’s exact test

^b^Significant differences (*P-*value < 0.05) are shown in bold

^c^ *OR* indicates odds ratio; 95% CI, 95% confidence interval

To further understand the role of mtDNA haplogroup on phenotypic expression for 1555A>G mutation, we evaluated the penetrance of hearing loss among 142 pedigrees (Table S3). A wide range of the penetrance of hearing loss were observed among the 142 pedigrees, with the average of 33.8% and 21.8% when aminoglycosides-induced hearing loss was included or excluded, respectively. Then the clinical penetrance of 1555A>G mutation on specific haplogroup were compared with that on all mtDNA backgrounds (Fig. 1). When aminoglycosides-induced hearing loss was included, we found 22 1555A>G pedigrees with haplogroup B background (50.7%), which is significantly higher than reference group (*P* = 0.006). Furthermore, when the aminoglycosides-exposed hearing loss cases were excluded, these haplogroup B pedigrees still showed significantly higher penetrance of hearing loss symptom (Average 35.6%, *P* = 0.0031).

**Figure 1:**
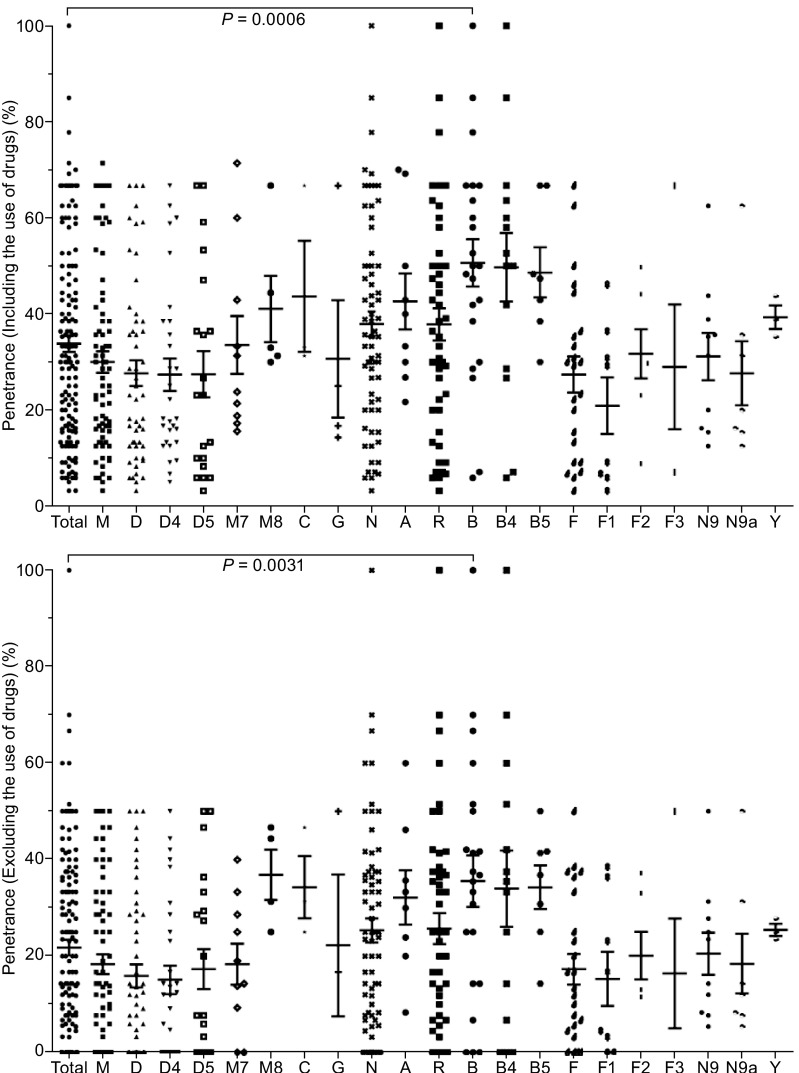
**Effect of mtDNA haplogroup on the penetrance of hearing loss in pedigrees with 1555A>G mutation**. *P* values were calculated by unpaired two tailed *t*-test. The haplogroups shared by at least three pedigrees were considered, and the M10 and M11 haplogroups were excluded. One family on haplogroup D was excluded since the lack of the penetrance of hearing loss. Five families were excluded since the lack of the penetrance of hearing loss without exposure to drugs

mtDNA haplogroups, as a product of ancient adaptation to environment, were formed during the evolution and migration of modern human and presented continental specific distributions (Wallace, 2005). According to the ancestral-susceptibility model, some mtDNA haplogroups may confer certain disease susceptibility, as well as the penetrance of the clinical phenotype, with the change of environment and lifestyle in modern time (Wallace, 2005). Nuclear–mitochondrial crosstalk is critical for the maintenance of cellular homeostasis. As the mtDNA haplogroups were characterized by distinct sets of variants, it is not surprising that certain mtDNA haplogroups could affect respiratory chain functions and may further perturb the crosstalk signaling. Indeed, a number of recent studies have revealed the associations between specific mtDNA haplogroups and various medical conditions such as Leber hereditary optic neuropathy, Parkinson disease, and Alzheimer’s disease (Hudson et al., 2007; Khusnutdinova et al., 2008; Malkki, 2013). The 1555A>G mutation is of multiple origins, and some specific haplogroups were found to be predominant among different ethnic backgrounds (Guan, 2011). Among Spanish pedigrees, the 1555A>G mutation resided at the mtDNA haplogroups H, I, J, K, T, U, V and L, and a strong excess of haplogroups H3 mtDNA carrying the 1555A>G mutation appeared in these families (Achilli et al., 2004). Here, we evaluate the contribution of mtDNA haplogroups to and expression of the hearing loss phenotype caused by 1555A>G mutation in Eastern Asian population.

Lineages M and N radiated to give rise to a plethora of mtDNA lineages in Asia. Here, the 142 pedigrees carrying the 1555A>G mutation were sporadically distributed among 11 major Asian mtDNAs haplogroups. Alternatively, in keeping with previous reports that the presence of 1555A>G mutation has occurred multiple times during evolution of mtDNA in Eastern Asia (Lu et al., 2010b). MtDNA haplogroup D, one of the most characteristic mtDNA lineages found in Northeast Asian populations, has been reported to be associated with various diseases. For instance, the specific haplogroup D, and its subgroup D4a and D5 might be susceptibility genetic background markers for breast cancer and esophageal cancer in Chinese population (Fang et al., 2010). In particular, a previous investigation suggested that haplogroup D4b and its subgroup D4b2 background has an increased risk of hearing loss in Japanese population (Kato et al., 2012). Here, we also found a significant excess of haplogroup D population comparing to the controls. There are some possible explanations associated with this observation. First, haplogroup D might increase the penetrance of 1555A>G mutation, thereby increasing the detection rate of this haplogroup in subjects with 1555A>G mutation. However, when we look into the penetrance of hearing loss in these 1555A>G mutation pedigrees, no statistic difference was observed comparing the haplogroup D population with others. Second, the high frequency of the 1555A>G mutation with haplogroup D might be the result of a founder effect. That is, one 1555A>G mutation occurred on a specific D mtDNA, and latter maternally transmitted by generations to generations with the hearing-impaired phenotype. In fact, 1555A>G mutation have also been reported to be an important founder event with H3 haplogroup among Spanish pedigrees (Achilli et al., 2004).

Instead, these subjects with haplogroup B background exhibited significantly higher penetrance of hearing loss. Haplogroup B is identified by a 9-bp deletion in the COII/tRNA^Lys^ intergenic region of mtDNA plus the HVS I motif 16189–16519. Previous studies have shown that the individuals on haplogroup B background are particularly susceptible to acute mountain sickness, and greatly increasing the severity of the clinical symptom in southwestern Han Chinese population (Li et al., 2011). In addition, the haplogroup B2 increased the risk for cervical cancer and showed an additive effect over the risk conferred by the human papillomavirus in Amerindian. In the present study, Pearson’s chi-square test revealed no significant difference of mtDNA haplogroup B prevalence between the 1555A>G and non-1555A>G populations. However, combination of the haplogroup B with the 1555A>G mutation significantly enhances the penetrance of hearing loss. Our results suggested that haplogroup B was associated with a significantly increased penetrance of hearing loss whenever aminoglycosides-induced hearing loss cases were included or excluded.

In conclusion, our finding suggest that the B haplogroup is associated with the increased penetrance of hearing loss phenotype in Asian population with 1555A>G mutation. The underlying mechanism is worthwhile to investigate further. Genotyping of these mtSNPs may provide information for predicting the genetic risk for hearing loss and may thereby contribute to the primary prevention of clinical syndrome in these population carrying 1555A>G mutation.

## Electronic supplementary material

Supplementary material 1 (PDF 688 kb)
