# Supplementary material for: Mitochondrial haplogroup B increases the risk for hearing loss among the Eastern Asian pedigrees carrying 12S rRNA 1555A>G mutation
Source: Protein Cell. 2015 Sep 11;6(11):844–8. doi: 10.1007/s13238-015-0203-z (PMC4624676; doi:10.1007/s13238-015-0203-z)
Supplement: Supplementary file 1 — Supplementary material 1 (PDF 688 kb) [file 13238_2015_203_MOESM1_ESM.pdf]

Materials and Methods

Subjects and Controls

A total of 11 Han Chinese pedigrees with non-syndromic hearing loss carrying 1555A>G mutation were collected by the Otology Clinic at the Wenling People's Hospital of Wenzhou Medical University, and all members with hearing loss were interviewed. Comprehensive examinations showed that hearing loss was the sole phenotype without any other clinical abnormalities. The classification of the severity was defined by using a pure-tone average at 500, 1000, 2000, 4000 and 8000Hz in the better hearing ear. Hearing impairment was classified as follows: normal hearing <26 Decibel(dB); mild hearing loss, 26-40dB; moderate hearing loss, 41-70dB; severe hearing loss, 71-90dB; and profound hearing loss, >90dB. In addition, 376 genetically unrelated Chinese subjects were enrolled as controls for the case-control association study. The age of the control subjects (193 males and 174 females) ranged from 8 to 58 years old, with an average of 21 years old. Furthermore, comprehensive history and physical examination showed these participants exhibited normal hearing and don't have a family history of hearing impairment. This study has been approved by the Ethics Committees of both Zhejiang University and Wenzhou Medical University, and written informed consent was obtained from all participants or their guardians (in the case of children). Additionally, the samples were collected anonymously.

mtDNA analysis

Total DNA was extracted from blood specimens by Puregene DNA Isolation Kit (Gentra Systems, Minneapolis, Minnesota, USA). The presence of the 1555A>G

mutation in 11 Han Chinese pedigrees was determined according to our previous study(Li et al., 2004). Furthermore, 24 overlapped fragments spanning the entire mtDNA sequences of 11 probands and 376 controls were amplified and sequenced as described elsewhere(Tang et al., 2007). Subsequently, the variants in mitochondrial genome were identified by comparing the revised Cambridge Reference Sequence (rCRS, NC_012920) with DNASTAR software program.

mtDNA haplogroup analysis

A total of 142 non-syndromic hearing loss pedigrees carrying 1555A>G mutation from Eastern Asia were recruited for mtDNA haplogroup analysis in this study. In addition to the 11 Han Chinese pedigrees mentioned above, 131 pedigrees were collected from the literature (See Table S2), consisted of 129 Chinese, 4 Japanese and 9 Korean families. The mtDNA complete sequences of 142 hearing loss subjects carrying 1555A>G mutation and 376 control subjects were assigned to the Asian mitochondrial haplogroups based on the PhyloTree database (http://www.phylotree.org). The classification tree of the entire mtDNAs of 142 pedigrees carrying the 1555A>G mutation was generated by haplogroup-diagnostic variants.

Data analysis

The differences in distribution of each haplogroup between hearing loss subjects with 1555A>G mutation and controls were assessed using Pearson's chi-square statistics and Fisher's exact test as appropriate. We evaluated the penetrance rates of hearing loss in the pedigrees with 1555A>G mutation on different haplogroup background

when the exposure of aminoglycosides were included or excluded, respectively. Then, the penetrance of each haplogroup was compared with all haplogroups as the reference group using unpaired two tailed *t*-test. The *P* value, odds ratio (OR), and 95% confidence intervals (CIs) were calculated. Unless indicated otherwise, a *P* value <0.05 was considered statistically significant. All statistical analyses were carried out using GraphPad Prism 5.0 (GraphPad Software, Inc., La Jolla, CA, USA).

References

Li, R., Greinwald, J.H., Jr., Yang, L., Choo, D.I., Wenstrup, R.J., and Guan, M.X. (2004). Molecular analysis of the mitochondrial 12S rRNA and tRNASer(UCN) genes in paediatric subjects with non-syndromic hearing loss. J Med Genet 41, 615-620.

Tang, X., Yang, L., Zhu, Y., Liao, Z., Wang, J., Qian, Y., Tao, Z., Hu, L., Wu, G., Lan, J., *et al.* (2007). Very low penetrance of hearing loss in seven Han Chinese pedigrees carrying the deafness-associated 12S rRNA A1555G mutation. Gene 393, 11-19.

Supporting Figure Legends

Figure S1. Eleven hearing-impaired pedigrees with 1555A>G mutation. The affected individuals are marked with filled symbols, and the arrows indicate the probands. Asterisks denote the individuals who had a history of exposure to aminoglycosides

Figure S2. Haplogroup distributions of 142 hearing-impaired pedigrees with 1555A>G mutation. The synonymous and nonsynonymous coding-region variants are denoted by “s” and “ns”, respectively. Variants in the transfer RNA and the ribosomal RNA genes are denoted by “t” and “r”, respectively. The variants in non-coding regions are indicated by “nc”.

Table S1. The mtDNA variants in eleven Chinese families with hearing loss.

Table S2. Summary of clinical and genetic characterization of 142 hearing-impaired pedigrees with 1555A>G mutation.

Table S3. Effect of mtDNA haplogroup on the penetrance of hearing loss in pedigrees with 1555A>G mutation.

Supporting Figure Legends

Figure S1. Eleven hearing-impaired pedigrees with 1555A>G mutation. The affected individuals are marked with filled symbols, and the arrows indicate the probands. Asterisks denote the individuals who had a history of exposure to aminoglycosides

Figure S2. Haplogroup distributions of 142 hearing-impaired pedigrees with 1555A>G mutation. The synonymous and nonsynonymous coding-region variants are denoted by "s" and "ns", respectively. Variants in the transfer RNA and the ribosomal RNA genes are denoted by "t" and "r", respectively. The variants in non-coding regions are indicated by "nc".

Table S1. The mtDNA variants in eleven Chinese families with hearing loss.

Table S2. Summary of clinical and genetic characterization of 142 hearing-impaired pedigrees with 1555A>G mutation.

Table S3. Effect of mtDNA haplogroup on the penetrance of hearing loss in pedigrees with 1555A>G mutation.

Figure S1

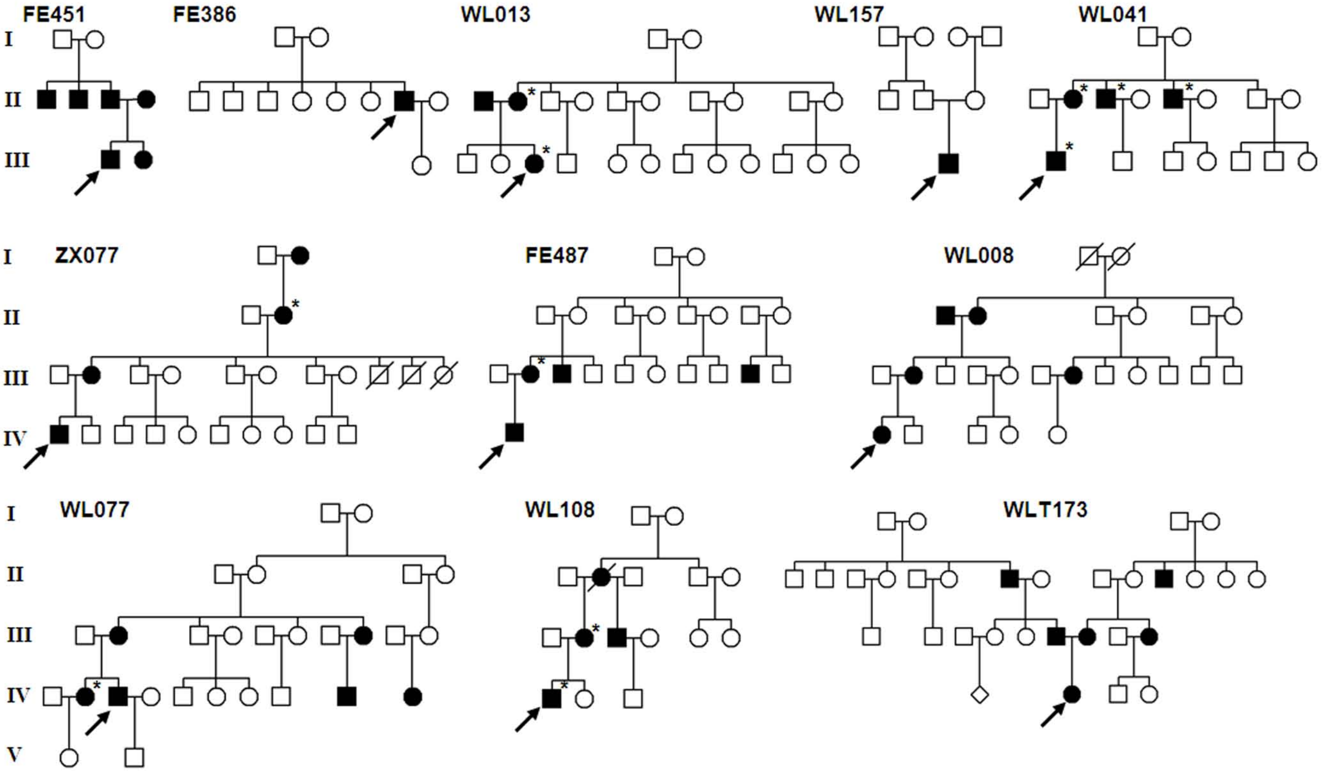

FE451
FE386
WL013
WL157
WL041
ZX077
FE487
WL008
WL077
WL108
WLT173
I
II
III
IV
V

Figure S2

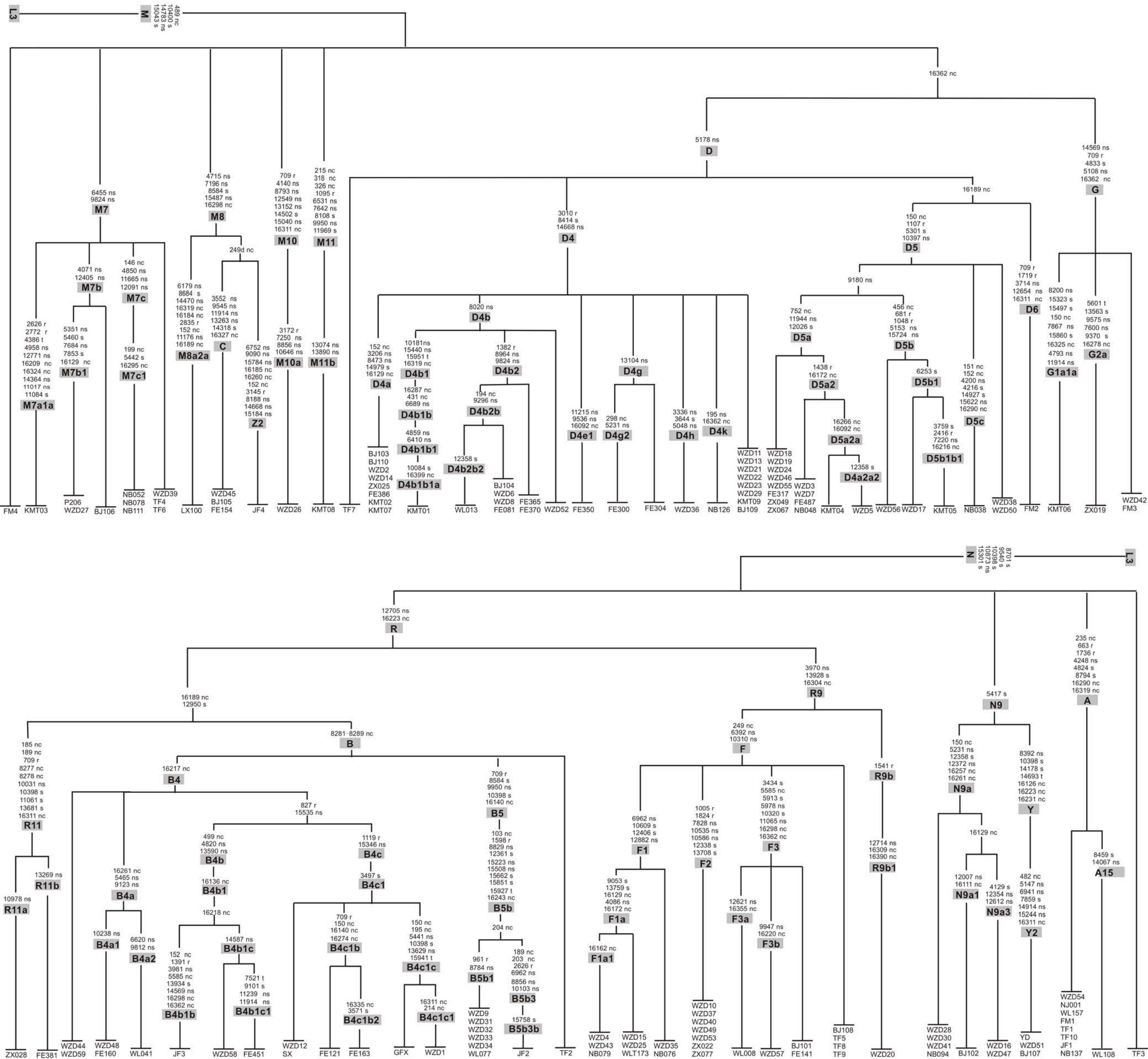

L3
M
M7
M7a1a
M7b
M7b1
M7c
M7c1
M8
M8a2a
C
Z2
M10
M10a
M11
M11b
D
D4
D4a
D4b
D4b1
D4b1b
D4b1b1
D4b1b1a
D4b2
D4b2b
D4b2b2
D4e1
D4g
D4g2
D4h
D4k
D5
D5a
D5a2
D5a2a
D4a2a2
D5b
D5b1
D5b1b1
D5c
D6
G
G1a1a
G2a
N
R
R9
F
F1
F1a
F1a1
F2
F3
F3a
F3b
R9b
R9b1
R11
R11a
R11b
B
B4
B4a
B4a1
B4a2
B4b
B4b1
B4b1b
B4b1c
B4b1c1
B4c
B4c1
B4c1b
B4c1b2
B4c1c
B4c1c1
B5
B5b
B5b1
B5b3
B5b3b
N9
N9a
N9a1
N9a3
Y
Y2
A
A15

Table S1 Summary of clinical and genetic characterization of 142 hearing-impaired pedigrees with 1555A>G mutation

Pedigree	Haplogroup	Number of matrilineal relatives	Penetrance (including the use of drugs) (%)	Penetrance (excluding the use of drugs) (%)	Reference
**Chinese (Mainland)**					
GFX	B4c1c		63.6	51.5	Bai Y, *et al*. 2010
P206	M7b1	7	71.4	28.6	Chen T, *et al*. 2013
NJ001	A	507	26.8	23.9	Li R， *et al*. 2003
SX	B4c1	19	52.6	42.1	Shen SS, *et al*. 2012
BJ101	F3	14	7.1	0.0	Young WY, *et al*. 2005
BJ102	N9a1	13	15.4	7.7	
BJ103	D4a	20	5.0	0.0	
BJ104	D4b2b	11	9.1	0.0	
BJ105	C	15	66.7	46.7	Zhao L, *et al*. 2005
BJ106	M7b	18	33.3	33.3	
BJ107	Y2	34	35.3	23.5	Young WY, *et al*.2006
BJ108	F2	16	62.5	37.5	
BJ109	D4	9	66.7	44.4	
BJ110	D4a	17	58.8	5.9	Yuan H, *et al*. 2005
WZD1	B4c1c1	17	5.9	0.0	Tang X, *et al*. 2007
WZD2	D4a	21	9.5	4.7	
WZD3	D5a2	16	12.5	0.0	
WZD4	F1a1	24	29.2	16.7	
WZD5	D5a2a2	31	3.2	0.0	
WZD6	D4b2b	8	25.0	12.5	
WZD7	D5a2	30	10.0	3.3	
WZD8	D4b2b	13	38.5	23.1	Chen B, et al. 2008
WZD9	B5b1	13	38.5	30.8	
WZD10	F2	8	50.0	37.5	
WZD31	B5b1	12	66.7	50.0	Wang X, *et al*. 2008
WZD32	B5b1	12	66.7	41.7	
WZD33	B5b1	29	48.3	41.4	
WZD34	B5b1	19	47.4	36.8	
WZD11	D4	19	52.6	42.1	Liao Z, *et al.* 2007
WZD12	B4c1	12.0	58.0	25.0	Lu J, *et al*. 2010
WZD13	D4	15	6.7	0.0	
WZD14	D4a	6	16.7	0.0	
WZD15	F1a	22	9.1	4.5	
WZD16	N9a3	16	31.3	31.3	
WZD17	D5b1	17	5.9	0.0	
WZD18	D5a	10	10.0	0.0	
WZD19	D5a	12	8.3	0.0	
WZD20	R9b1	16	12.5	0.0	
WZD21	D4	25	16.0	12.0	
WZD22	D4	29	41.4	31.0	
WZD23	D4	22	18.2	13.6	
WZD24	D5a	11	36.4	36.4	
WZD25	F1a	13	30.8	23.1	
WZD26	M10a	6	33.3	33.3	
WZD27	M7b1	7	42.9	0.0	

WZD28	N9a	24	12.5	8.3	
WZD29	D4	8	12.5	0.0	
WZD30	N9a	8	62.5	50.0	
WZD35	F1	15	6.7	0.0	
WZD36	D4 h	15	13.3	0.0	
WZD37	F2	30	30.0	20.0	
WZD38	D5	22	59.1	50.0	
WZD39	M7	16	18.8	0.0	
WZD40	F2	20	30.0	25.0	
WZD41	N9a	37	16.2	5.4	
WZD42	G	7	14.3	0.0	
WZD43	F1a1	62	3.2	3.2	
WZD44	B4	14	7.1	0.0	
WZD45	C	12	33.0	25.0	
WZD46	D5a	14	35.7	28.6	
WZD47	N9a3	25	20.0	12.0	
WZD48	B4a1	15	26.7	6.7	
WZD49	F2	30	23.3	13.3	
WZD50	D5	15	53.3	46.7	
WZD51	Y2	18	38.9	27.8	
WZD52	D4b	19	15.8	0.0	
WZD53	F2	17	35.3	11.8	
WZD54	A	25	40.0	20.0	
WZD55	D5a	15	13.3	0.0	
WZD56	D5b	17	47.1	29.4	
WZD57	F3b	20	20.0	10.0	
WZD58	B4b1c	8	50.0	37.5	
WZD59	B4	31	41.9	35.5	
YD	Y2	16	43.8	25.0	Ding Y, *et al*. 2009
FE081	D4b2b	17	17.6	11.8	Zhang T, *et al*. 2011
FE122	B4c1b	6	50.0	33.3	
FE141	F3	6	66.7	50.0	
FE154	C	16	31.3	31.3	
FE317	D5a	12	23.1	7.7	
FE160	B4a1	5	60.0	60.0	Peng G, *et al*. 2012
FE163	B4c1b2	20	85.0	70.0	
FE300	D4g2	14	35.7	28.6	
FE304	D4g	7	28.6	14.3	
FE350	D4e1	6	16.7	16.7	
FE365	D4b2	13	38.5	38.5	
FE370	D4b2	11	18.2	9.1	
FE381	R11b	10	20.0	20.0	
NB038	D5c	15	26.7	20.0	
NB048	D5a2	17	5.9	5.9	
NB052	M7c1	21	23.8	19.0	
NB076	F1	17	5.9	0.0	
NB078	M7c1	29	17.2	13.8	
NB079	F1a1	13	46.1	38.5	
NB094	N9a	14	35.7	14.3	
NB111	M7c1	32	15.6	9.3	
NB126	D4k	12	33.3	25.0	

NB137	A	10	30.0	30.0	
LX100	M8a2a	9	44.4	44.4	
ZX019	G2a	12	16.7	16.7	
ZX022	F2	11	9.1	0.0	
ZX025	D4a	9	22.2	0.0	
ZX028	R11a	23	39.1	34.8	
ZX049	D5a	13	23.1	7.7	
ZX067	D5a	17	5.9	0.0	
ZX077	F2	11	44.4	33.3	
WL008	F3a	18	22.2	5.6	
WL013	D4b2b2	15	13.3	0.0	
WL041	B4a2	6	66.7	0.0	
WL077	B5b1	7	42.9	14.3	
WL108	A15	14	42.9	35.7	
WLT173	F1a	11	36.4	36.4	
FE386	D4a	8	12.5	0.0	
FE451	B4b1c1	3	100.0	100.0	
FE487	D5a2	11	36.4	27.3	
WL157	A	3	33.3	33.3	
FM1	A	23	21.7	/	Liu C, *et al*. 2010
FM2	D6	5	40.0	/	
FM3	G	8	25.0	/	
FM4	M*	19	21.1	/	
**Chinese (Taiwan)**					
TF1	A	13	69.2	46.2	Wu CC, *et al*. 2007
TF2	B	9	77.8	66.7	
TF3	N*	3	66.7	0.0	
TF4	M7	14	21.4	14.3	
TF5	F	15	13.3	6.7	
TF6	M7	16	31.3	25.0	
TF7	D	20	30.0	10.0	
TF8	F	6	33.3	16.7	
TF9	F	13	15.4	7.7	
TF10	A	12	50.0	8.3	
**Japanese**					
JF1	A	10	70.0	60.0	Yamasoba T, *et al*. 2002
JF2	B5b3b	20	30.0	25.0	
JF3	B4b1b	14	28.6	14.3	
JF4	Z2	110	30.0	/	Matsunaga T, *et al*. 2005
**Korean**					
KMT01	D4b1b1a	8	62.5	50.0	Bae JW, *et al*. 2002
KMT02	D4a	5	60.0	40.0	
KMT03	M7a1a	5	60.0	40.0	
KMT04	D5a2a	3	66.7	33.3	
KMT05	D5b1b1	6	66.7	50.0	
KMT06	G1a1a	6	66.7	50.0	
KMT07	D4a	7	28.6	14.3	
KMT08	M11b	5	60.0	40.0	
KMT09	D4	/	/	/	

Table S2 The mtDNA variants in eleven Chinese families with hearing loss

Gene	Position	Replacement	ZX077	WL008	WL013	WL041	WL077	WL108	WL157	WLT173	FE386	FE451	FE487	Previously reported
DLOOP	73	A to G	G	G	G	G	G	G	G	G	G	G	G	Yes
	103	G to A					A							Yes
	146	T to C							C					Yes
	150	C to T											T	Yes
	152	T to C						C		C	C			Yes
	194	C to T			T									Yes
	199	T to C				C								Yes
	204	T to C		C										Yes
	207	G to A or C		A				A						Yes
	235	A to G						G	G					Yes
	249	del A	del A	del A						del A				Yes
	263	A to G	G	G	G	G	G			G	G	G	G	Yes
	293	T to C							C					Yes
	310	T to TC or CTC or CCTC or CCCTC	CCTC	CCTC	TC	CCTC	CCTC	TC	CTC	CTC	CTC	CTC		Yes
	489	T to C			C						C			Yes
	499	G to A										A		Yes
	521-524	del ACAC								del ACAC				Yes
	523	del A			del A	del A	del A	del A	del A				del A	Yes
	524	del C			del C	del C	del C	del C	del C				del C	Yes
	16093	T to C									C			Yes
	16129	G to A								A	A			Yes
	16136	T to C										C		Yes
	16140	T to C					C							Yes
	16164	A to G											G	Yes
	16172	T to C								C			C	Yes
	16182	A to C				C							C	Yes
	16183	A to C/G				C	C					G	C	Yes
	16189	T to C or CC				C	C		C			C	C	Yes
	16193	T to CC					CC					CC		Yes
	16217	T to C				C						C		Yes
	16218	C to T										T		Yes
	16223	C to T			T			T	T		T		T	Yes
	16243	T to C					C							Yes
	16259	C to T											T	Yes
	16260	C to T		T										Yes
	16261	C to T				T								Yes
	16263	T to C									C			Yes
	16290	C to T						T						Yes
	16298	T to C/T		C										Yes
	16304	T to C/G	C							C				Yes
	16310	A to G				G								Yes
	16311	T to C					C							Yes
	16319	G to A						A	A					Yes
	16355	C to T		T			T							Yes
	16360	C to T				T								Yes
	16362	T to C		C	C			C			C		C	Yes
	16519	T to C			C		C			C		C		Yes
	16527	C to T	T								T			Yes
MT-RNR1	663	A to G						G	A					Yes
	709	G to A		A			A							Yes
	750	A to G	G	G	G	G	G	G	G	G	G	G	G	Yes
	752	C to A/T											T	Yes
	827	A to G										G		Yes
	872	G to A						A						Yes
	961	T to CC					CC							Yes
	1005	T to C	C											Yes
	1107	T to C											C	Yes
	1382	A to G			G									No
	1438	A to G	G	G	G	G	G	G	G	G	G	G		Yes
	**1555**	**A to G**	G	G	G	G	G	G	G	G	G	G	G	Yes
	1598	G to A					A							Yes
MT-RNR2	1736	A to G						G	G					Yes
	1824	T to C	C											Yes

	2280	C to A	A											Yes
	2281	A to G	G											Yes
	2706	A to G	G	G	G	G	G	G	G	G	G	G	G	Yes
	2766	C to T							T					Yes
	3010	G to A	A		A						A			Yes
	3107	del N	del N	del N	del N	del N	del N	del N	del N	del N	del N	del N	del N	Yes
	3202	T to C										C		Yes
	3206	C to T									T			Yes
MT-ND1	3357	G to A	A											Yes
	3396	T to C								C				Yes
	3434	A to G(Thr to Cys)		G										Yes
	3552	T to A											A	Yes
	3591	G to A						A						Yes
	3849	G to A							A					Yes
	3866	T to C(Ile to Thr)					C							Yes
	3970	C to T	T	T						T				Yes
	4086	C to T								T				Yes
	4248	T to C						C	C					Yes
MT-TI	4317	A to G												Yes
MT-TQ	4387	C to T												No
MT-ND2	4715	A to G											G	Yes
	4733	T to C								C				Yes
	4740	A to G(Asn to Lys)						G						Yes
	4769	A to G	G		G	G	G	G	G	G	G	G	G	Yes
	4820	G to A										A		Yes
	4824	A to G(Thr to Ala)						G	G					Yes
	4883	C to T			T						T			Yes
	5040	A to G(Met-Val)				G								Yes
	5178	C to A(Leu to Met)			A						A			Yes
	5237	G to A						A						Yes
	5465	T to C				C								Yes
MT-NC3	5585	G to A		A										Yes
MT-NC5	5894	A to G		G										Yes
MT-CO1	5913	G to A(Asp to Asn)		A										Yes
	5978	A to G		G										Yes
	6026	G to A											A	Yes
	6392	T to C	C	C						C				Yes
	6620	T to C				C								Yes
	6962	G to A								A				Yes
	7028	C to T	T	T	T	T	T	T	T	T	T	T	T	Yes
	7196	C to A											A	Yes
MT-TS1	7521	C to T										T		Yes
MT-CO2	7828	A to G	G											Yes
	7999	T to C											C	Yes
	8020	G to A			A									Yes
MT-NC7	8281_8289	9-bp del				9-bp del	9-bp del					9-bp del		Yes
MT-ATP8	8414	C to T(Leu to Phe)			T						T			Yes
	8459	A to G(Asn to Asp)						G						Yes
	8473	T to C									C			Yes
MT-ATP6	8584	G to A(Ala to Thr)					A						A	Yes
	8701	A to G (Thr to Ala)			G						G		G	Yes
	8784	A to G					G							Yes
	8794	C to T(His to Tyr)						T	T					Yes
	8829	C to T					T							Yes
	8860	A to G(Thr to Ala)	G	G	G	G	G	G	G	G	G	G	G	Yes
	8964	C to T			T									Yes
	9053	G to A(Ser to Asn)			A					A				Yes
	9101	T to G(Ile to Ser)										G		Yes
	9123	G to A				A								Yes
	9180	A to G											G	Yes
MT-CO3	9296	C to T			T									Yes
	9540	T to C			C						C		C	Yes
	9548	G to A				A		A		A				Yes
	9812	C to T				T								Yes
	9824	T to A/C			A									Yes
	9845	T to C									C			Yes
	9854	T to C		C										Yes
	9861	T to C(Phe to Leu)										C		Yes
	9950	T to C					C							Yes

MT-ND3	10265	T to C	C														Yes
	10310	G to A	A	A						A							Yes
	10320	G to A(Val to Ile)		A													Yes
	10397	A to G													G		Yes
	10398	A to G (Thr to Ala)			G		G				G				G		Yes
	10400	C to T			T						T				T		Yes
MT-ND4L	10499	A to G		G													Yes
	10535	T to C	C														Yes
	10586	G to A	A														Yes
	10609	T to C (Met to Thr)								C							Yes
MT-ND4	10873	T to C			C						C				C		Yes
	10876	A to G				G											Yes
	11065	A to G		G													Yes
	11084	A to G(Thr to Ala)						G									Yes
	11152	T to C						C									Yes
	11239	A to G											G				Yes
	11719	G to A	A	A	A	A	A	A	A	A		A		A		A	Yes
	11914	G to A											A				Yes
	11944	T to C													C		Yes
	12026	A to G (Ile to Val)													G		Yes
MT-ND5	12338	T to C(Met to Thr)	C														Yes
	12358	A to G(Thr to Ala)			G												Yes
	12361	A to G(Thr to Ala)					G										Yes
	12406	G to A (Val to Ile)								A							Yes
	12621	C to T		T													Yes
	12705	C to T			T			T	T		T					T	Yes
	12879	T to C									C						Yes
	12882	C to T								T							Yes
	13105	A to G(Ile toVal)				G											Yes
	13290	C to T											T				Yes
	13329	C to T									T						Yes
	13569	T to C						C									Yes
	13590	G to A											A				Yes
	13602	T to C	C														Yes
	13708	G to A (Ala to Thr)	A					A									Yes
	13759	G to A (Ala to Thr)								A							Yes
	13928	G to C/A (Ser to Thr)	C	C						C							Yes
	14067	C to T						T									Yes
MT-ND6	14323	G to A									A						Yes
	14560	G to A					A										Yes
	14587	A to G											G				Yes
	14668	C to T									T						Yes
MT-CYB	14766	C to T(Thr to Ile)	T	T		T	T	T	T	T		T		T		T	Yes
	14769	A to G(Asn to Ser)	G														Yes
	14783	T to C									C				C		Yes
	14842	C to A(Asn to Lys)			A												No
	14971	T to C		C													Yes
	14979	T to C(Ile to Thr)									C						Yes
	15043	G to A			A						A				A		Yes
	15223	C to T					T										Yes
	15301	G to A			A						A				A		Yes
	15326	A to G (Thr to Ala)	G	G	G	G	G	G	G	G		G		G		G	Yes
	15373	A to G								G							Yes
	15508	C to T					T										Yes
	15535	C to T											T				Yes
	15662	A to G(Ile to Val)					G										Yes
	15851	A to G(Ile to Val)					G										Yes
MT-TT	15889	T to C									C						Yes
	15924	A to G						G					G				Yes
	15927	G to A					A										Yes
	15943	T to C		C													Yes

Table S3 Effect of mtDNA haplogroup on the penetrance of hearing loss in pedigrees with 1555A>G mutation.

Haplogroup[a]	Average penetrance (including the use of drugs)[b]	*P* Value[c]	Average penetrance (excluding the use of drugs)[d]	*P* Value
M	30.0	0.9110	18.3	0.2041
D	27.7	0.0660	15.9	0.0545
D4	27.4	0.1200	15.1	0.0792
D5	27.5	0.2021	17.3	0.3270
M7	33.6	0.9710	18.3	0.5744
M8	41.1	0.4391	36.9	0.1176
C	43.7	0.4157	34.3	0.2576
G	30.7	0.7653	22.2	0.9677
N	37.9	0.1886	25.3	0.2231
A	42.7	0.2126	32.2	0.1322
R	37.8	0.2625	25.7	0.2377
**B**	**50.7**	**0.0006**	**35.6**	**0.0031**
**B4**	**49.7**	**0.0084**	34.0	0.1529
B5	48.6	0.0632	34.3	0.0877
F	27.4	0.1614	17.3	0.2830
F1	20.9	0.0855	15.3	0.3467
F2	31.7	0.7915	20.1	0.8209
F3	29.0	0.6481	16.4	0.5795
N9	31.2	0.6905	20.5	0.8388
N9a	27.7	0.4398	18.4	0.6480
Y	39.3	0.6464	25.4	0.0997
Average	33.8	-	21.8	-

a. The haplogroups shared by at least three pedigrees were considered.
b. Qne family on haplogroup D was excluded since the lack of the penetrance of hearing loss.
c. *P* values were calculated by unpaired two tailed *t*-test.
d. Five families were excluded since the lack of the penetrance of hearing loss without exposure to drugs.
e. Significant differences (*P* value<0.05) are shown in bold.
